# Reptiles in Space Missions: Results and Perspectives

**DOI:** 10.3390/ijms20123019

**Published:** 2019-06-20

**Authors:** Victoria Gulimova, Alexandra Proshchina, Anastasia Kharlamova, Yuliya Krivova, Valery Barabanov, Rustam Berdiev, Victor Asadchikov, Alexey Buzmakov, Denis Zolotov, Sergey Saveliev

**Affiliations:** 1 Research Institute of Human Morphology, Ministry of Science and Higher Education RF, Tsurupi street, 3, 117418 Moscow, Russia; proshchina@ya.ru (A.P.); grossulyar@gmail.com (A.K.); homulkina@rambler.ru (Y.K.); valbaraban@yandex.ru (V.B.); braincase@yandex.ru (S.S.); 2 Research and Educational Center for Wild Animal Rehabilitation, Faculty of Biology, M.V. Lomonosov Moscow State University, Leninskie Gory, 1/12, 119899 Moscow, Russia; rberdiev@gmail.com; 3 Shubnikov Institute of Crystallography of FSRC “Crystallography and Photonics”, Russian Academy of Sciences, Leninsky Ave, 59, 119333 Moscow, Russia; asad@crys.ras.ru (V.A.); buzmakov@gmail.com (A.B.); zolotovden1985@gmail.com (D.Z.)

**Keywords:** spaceflight adaptation, unmanned spacecraft, Foton-M2, Foton-M3, Bion-M1, Foton-M4, thick-toed gecko (*Chondrodactylus turneri*), ornate day gecko (*Phelsuma ornata*), X-ray microtomography

## Abstract

Reptiles are a rare model object for space research. However, some reptile species demonstrate effective adaptation to spaceflight conditions. The main scope of this review is a comparative analysis of reptile experimental exposure in weightlessness, demonstrating the advantages and shortcomings of this model. The description of the known reptile experiments using turtles and geckos in the space and parabolic flight experiments is provided. Behavior, skeletal bones (morphology, histology, and X-ray microtomography), internal organs, and the nervous system (morphology, histology, and immunohistochemistry) are studied in the spaceflight experiments to date, while molecular and physiological results are restricted. Therefore, the results are discussed in the scope of molecular data collected from mammalian (mainly rodents) specimens and cell cultures in the parabolic and orbital flights and simulated microgravity. The published data are compared with the results of the gecko model studies after the 12–44.5-day spaceflights with special reference to the unique peculiarities of the gecko model for the orbital experiments. The complex study of thick-toed geckos after three spaceflights, in which all geckos survived and demonstrated effective adaptation to spaceflight conditions, was performed. However, future investigations are needed to study molecular mechanisms of gecko adaptation in space.

## 1. Introduction

Space research into animal models may provide helpful information for the human space mission organization and data on the adaptive scopes and perspectives of various species. Space studies aboard unmanned satellites and space stations are unique as it is difficult to simulate prolonged spaceflight conditions, including weightlessness (G_0_), on Earth. To date, the most popular model objects for space research projects have been rodent specimens [1,2,3,4,5]. The adaptive features, including different mechanisms from molecular pathways to cognitive and behavioral adaptations, to the space condition of the exact species are determined by the biology and phylogenetic history of the species [6]. There have also been numerous studies on fish models [7,8,9,10,11,12]; amphibians, such as clawed frogs [13] and ribbed newts [14,15,16,17,18]; and birds (Japanese quail) [19,20,21,22,23,24]. Reptiles are rare model objects for space and microgravity research [25,26,27,28,29,30,31,32,33,34] (Table 1). Before the gecko experiments, only the steppe tortoises *Testudo horsfieldii* were used for the 7-day spaceflights aboard the Zond 5 and Zond 7 satellites (USSR) [27] (Table 1). They had also flown for 19, 22, 60, and 90 days [28]. Turtles [25,35] and 23 other species of amphibians and reptiles, including geckos [26], were studied in parabolic flights with short-term (7 and 20–25 s) weightlessness (Table 1). However, not all results of the studies with parabolic flight short-term microgravity were confirmed in long-term space experiments. Parabolic flights were interpreted as a method for the study of the reaction to changing gravity, but not of weightlessness itself [36].

The main problems of mammalian adaptation to spaceflight are flotation and restricted mobility in most types of containers used for space research. Both are stress factors, the influence of which is difficult to separate from the effect of weightlessness itself. All reptiles previously used for space researches floated under spaceflight conditions, as did mammals and amphibians [26,36], with the exception of cases when rodents (mice and gerbils) could move, or even run, clinging to the grating of the container [5].

In normal Earth gravity, many geckos are able to attach to the surfaces oriented in almost any way by means of the setae on geckos’ subdigital pads [37,38]. This favorably distinguishes the geckos, for example, from the ribbed newts (*Pleurodeles waltl*, Michahelles, 1830), another model object of space researches widely used in the past [16,18]. The ability of thick-toed geckos to remain attached to smooth surfaces during weightlessness was clearly shown [26,31,32], although for a long time gravity had been assumed to be required for this [39,40]. This peculiar ability allows the geckos to keep normal activities and behavior during weightlessness [41,42].

To date, geckos have been used in a number of orbital experiments, in which the high ability of geckos to adapt to spaceflight conditions has been shown. The researches after 16- and 12-day orbital experiments onboard the unmanned spacecraft (USC) series Foton-M2 and Foton-M3, respectively, as well as after a 30-day flight on USC Bion-M1 included

(i) histological staining of internal organs [29,33,43,44];
(ii) immunohistochemistry [33,44];
(iii) X-ray Absorption Microtomography (XAM) [31,32,45,46];
(iv) scanning Electron Microscopy (SEM) [45];
(v) X-ray Fluorescence Analysis (XFA) [45,46]; and
(vi) behavioral studies (Foton-M3, Foton-M4, and Bion-M1 only) [6,32,34,41,42].

Reptile experimental objects used for spaceflight studies to-date (turtles and geckos) are easy-handled animals and possess a tolerance for water and food absence during spaceflight, which is very useful for the prolonged experiments onboard unmanned satellites. Also, wide diversity in the ecology and biology of gecko species allows scientists to choose optimal specimens for easy maintenance of the experimental design. The primary scope of the review is to demonstrate the benefits and limitations of the gecko model for the further space investigations in comparison with other reptiles and mammals, which had been widely used for spaceflight experiments.

In this review, the results of gecko spaceflight experiments, such as behavioral adaptations; skeletal bones morphology, histology, and X-ray microtomography; and internal organ and nervous system morphology; histology, and immunohistochemistry, are discussed with special reference to the unique peculiarities of the gecko model for the orbital experiments. These results are also compared with the molecular data collected from mammalian (mainly rodents) specimens and cell cultures in the parabolic and orbital flights and simulated microgravity.

## 2. Behavioral Adaptations of Geckos to Spaceflight Conditions

The behavior of reptiles in weightlessness is poorly studied. In parabolic flights, it was first investigated by von Beckh in 7-second parabolas [25] on South American aquatic turtles *Trachemys ornata* and *Hydromedusa tectifera* (Table 1). His result suggests that in weightlessness, animals can reorient from vestibular to other stimuli, for example, visual stimuli. From the 1990s to 2000s behavioral studies in parabolic flights were continued [26,35] (Table 1). The behavior of the 21th reptile species in weightlessness lasted for 20–25 s was documented by means of video registration. The leaf-tail gecko *Uroplatus* was reported to be in contact with container surfaces during weightlessness, but without any explication whether it was actively adhering to or passively in contact with the surfaces [26].

**Figure 1:**
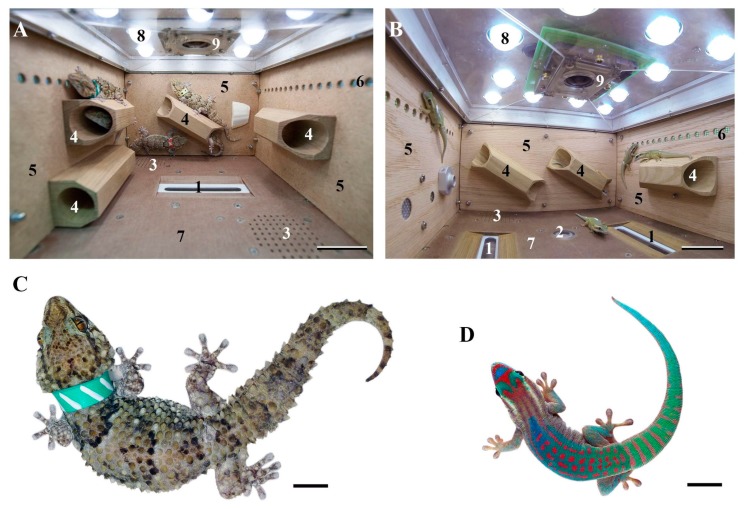
The research and support block (RSB) and geckos used for orbital experiments. (**A**) RSB with thick-toed geckos (Bion-M1 satellite, 30-day spaceflight). (**B**) RSB with ornate day geckos (Foton-M4 satellite, 44,5-day spaceflight). (**C**) Thick-toed gecko female with color marking collar. (**D**) Ornate day gecko male. 1—feedbox; 2—water bowl; 3—heating zones; 4—tubular shelters for geckos made of American oak; 5—RSB walls lined with (**A**) hardboard (a type of fiberboard); (**B**) American oak; 6—vents for ventilation and waste collection; 7—RSB floor made of textile laminate (a fabric reinforced laminate); 8—LEDs; 9—video camera and a fan. Only the RSB for ornate day geckos was equipped with feedbox and water bowl. For a more detailed description of the life support system, see [41]. Bar: (**A**) 5 cm, (**B**) 5 cm, and (**C**,**D**) 1 cm.

The behavior of two species of reptiles: nocturnal thick-toed geckos *(Chondrodactylus turneri)* and ornate day geckos (*Phelsuma ornata*) was studied in long-term orbital experiments (Figure 1). The main data of these experiments are in the Table 1 and Table 2.

Behavioral analysis of video recordings was made after 12- and 30-day flights with thick-toed geckos [41,42] and 44.5-day flights with ornate day geckos [34]. The enzyme-linked immunosorbent assay (ELISA) study of excrements was spent only after 12- and 16-day flights [32] and cell cycle analysis—only after 16-day flight [30].

Video recordings from 12-day spaceflight demonstrated for the first time that thick-toed geckos in weightlessness retain the ability to attach to surfaces [31]. More frequent head movements registered in the resting geckos during the 12-day spaceflight onboard Foton-M3 were suggested to be caused by the animal trying to stimulate the vestibular apparatus using head movements [6].

A study of thick-toed gecko behavior during a 30-day orbital experiment revealed that, on average, the geckos spent 99.9% of the time adhering to surfaces during the flight and only 0.1% floating freely. The responses during flotation were similar to the behavioral responses to falling under normal gravity, including the ventral extension of the limbs, skydiving posture, and postural righting reflexes. It was also speculated that the ability of geckos to keep attached to surfaces is an important factor of the animal adaptation to weightlessness [42].

Thick-toed geckos object play behavior was firstly described in the same 30-day orbital experiment onboard Bion-M1. The geckos played with a collar that was removed by one of them in the prelaunch period and that floated under microgravity. Explanations for the rarity of play behavior in reptiles under normal conditions and the geckos’ playfulness in microgravity were discussed and the high adaptive capability of geckos for the space conditions was concluded [41].

For the 60-day experiment on the USC Foton-M4, the ornate day geckos (*Phelsuma ornata)* was chosen mainly because they need not live insects for food. It was found that ornate day geckos, worse than thick-toed geckos, can control paw adhesiveness during movement. On the other hand, the ability of ornate day geckos to jump, even in the first few minutes of weightlessness, was better. 

Thus, the geckos demonstrated the high adaptive behavior in the weightlessness during spaceflight aboard unmanned satellites. The play behavior of thick-toed geckos in weightlessness also testifies to their well-being and the absence of chronic stress [47]. The behavioral study results coincide with the data on reversible cytomorphological changes in Purkinje cells of thick-toed geckos and mice after spaceflight (see below) [44]. At the same time, the preliminary results with ELISA for the corticosterone of the thick-toed geckos’ excrements from the flight and synchronous control groups in the Foton-M2 and Foton-M3 were ambiguous [32]. Corticosterone level in the flight group geckos in the Foton-M2 was 4 times bigger than in the synchronous control group. Geckos from the flight group of the Foton-M3 experiment have no significant difference in comparison to the synchronous control group. Moreover, it was close to the level of the synchronous control group geckos from the Foton-M2 experiment. A high level of the hormone in the flight group Foton-M2 geckos could be related to the time of transportation from space center Baikonur to the laboratory after landing (32 h in Foton-M2 and 13.5 h in Foton-M3). Also it is known that increased corticosterone level reflects a stress caused by the multiple factors, including water–salt metabolism changes.

The adhesion ability allowed thick-toed and ornate day geckos to stay attached and maintain normal locomotion for the major part of the flights (up to 99.9% of flight time (12–45 days)), thus avoiding the stress caused by floating [6,32,42]. The used gecko species are also perspective model for the studies aboard unmanned spacecraft due to their small size, resistance to fasting and absence of water, and relatively low aggression.

## 3. Gecko Brain in Prolonged Spaceflight Experiments

It has been well established that environmental manipulation may lead to anatomical, physiological, and chemical changes in the brain [48]. In humans, general neurological changes including space motion sickness, postural illusions, visual disturbances, cephalic fluid shifts, cognitive alterations, neuromuscular fatigue and weakness, as well as postural imbalance and ataxia upon return to Earth are seen during and following spaceflight [49,50,51,52,53]. Microgravity has been suggested as a reason for space-related neurologic dysfunction. In mammals, microgravity is believed to induce apoptosis and affect signal transduction cell pathways, which change cell proliferation and differentiation, migration, and adhesion [54], due to gravity contributing to the spatial relationship of the intracellular organelles and cytoskeleton [55]. Consequently, DNA replication, RNA transcription, and protein transport and synthesis could also be affected [56]. According to published data, alterations in many gene activation and gene pathway changes and those associated with the influence of spaceflight conditions have been identified [57]. Moreover, microgravity, and possibly other nonspecific effects of spaceflight, can alter normal brain development [58]. There are also data showing that space-relevant radiation may induce changes in neuronal functions. Additionally, exposure to spaceflights has a strong impact on metabolic and stress response [59].

Involvement of the cerebellum, cortical sensorimotor and somatosensory areas, and the vestibular pathways of the brainstem in brain alterations caused by spaceflight, were studied most frequently [51,52,60,61].

Before the Foton-M series, there were no reptile brain studies in the space condition. It was only the peripheral nervous system study of turtles after the 6.5-day orbital experiment performed [27]. As in previous studies of the vertebrate brain, changes in the cerebral cortex, cerebellum, and brainstem vestibular nuclei were observed after spaceflight, and an in-depth analysis of these structures in geckos was performed.

In general, the histological structure of the brain was normal in all geckos which were flown on-board Foton-M2, Foton-M3, and Bion-M1 satellites. However, reversible cytomorphological changes (such as chromatolysis, vacuolization, and hyperchromatosis) were detected in some brain cells. Such pathomorphological cell changes correspond to the high metabolic activity of cell. These nonspecific alterations were revealed in the Purkinje cells of the cerebellum [44], neurons of the medial and dorsomedial cortex, and the cell bodies of vestibular nuclei of the rhombencephalon (nucleus vestibularis ventrolateralis (Vevl), nucleus vestibularis descendens, and nucleus vestibularis dorsolateralis) [43].

Intensive vacuolization was shown in the peripheral cytoplasm of the vestibular neurons bodies. These changes were mostly expressed in the large neurons of the Vevl. At the same time, no such cytological changes were revealed in the neurons of the adjacent acoustic area of the medulla oblongata. It can be assumed that these cytomorphological features are a manifestation of the in vivo state of neurons, which correlates with the level of metabolism. 

In humans and other vertebrates, spaceflight conditions cause alterations in vestibular and motor functions, including changes in linear vestibular-ocular reflexes and postural control systems. These changes induce posture and visual perception illusions, illusions of head movements and rotation, nystagmus, and vertigo and space motion sickness symptoms [62,63]. In the central nervous system, vestibular inputs are correlated with visual and proprioceptive signals to control motions and compensate head and eye movements [64]. Therefore, changes in the linear acceleration perception system are primarily expected during long-term spaceflights. Alterations in the Purkinje cell layer of cerebellum are the most frequently observed in spaceflight and altered gravity conditions [65,66,67,68]. In addition, the morphology of the reptilian cerebellum is similar to the basic cerebellar structure of terrestrial vertebrates.

A quantitative analysis of the number of Purkinje cells in anterior and posterior (vestibular) cerebellum after Bion-M1 was performed. A significant increase in the number of Purkinje cells with cytomorphological changes, described above, was revealed only in the posterior (vestibular) cerebella of the geckos from the flight group in comparison with the control groups. It is reasonable to speculate that these changes, detected in the vestibular cerebellum Purkinje cells of geckos, were also caused by functional loading on the cells of this type during the spaceflight [44]. Moreover, similar data were obtained from the Purkinje cell study using neuron-specific beta-III-tubulin (NST) immunohistochemistry. An increase in the number of Purkinje cells with an altered NST immunoreactivity was revealed in the flight group. There were no significant differences in the distribution and NST immunoreactivity of Purkinje cells in the anterior cerebellum between the flight and control groups of geckos. The density of Purkinje cell dendrites was also measured. There were no statistically significant alterations in dendrite density between the groups of geckos in both the anterior and posterior cerebellum [69].

These data coincide with gecko behavior during spaceflight, which was described in the Bion-M1 space mission project (see above). It was proposed that the nervous system is able to compensate for incorrect information from the vestibular system, using tactile and visual signals. This hypothesis is confirmed in the recently published study of the behavior of mice aboard the International Space Station [5]. In this study, video images were acquired during orbit from 16- and 32-week-old female mice. Younger (but not older) mice began to exhibit “race-tracking” behavior that evolved into coordinated group activity within 7 to 10 days after launch. This behavior unique to spaceflight may represent stereotyped motor behavior, rewarding the effects of physical exercise, or vestibular sensation produced via self-motion. The authors of this study have remarked that “vestibular self-stimulation would involve activity within the otolith organs and central nuclei of the balance system and may overlap with activation of stress and/or reward pathways.”

The hippocampus is another structure to which much attention is paid in many studies on spaceflight effects [55]. The reptilian medial and dorsomedial cortex is a homologue of the hippocampus of mammals. 

Antibodies to the NGF-receptor (p75NGFR) and CD95 (also known as Fas) were used for the detection of neurodegenerative changes in brain of thick-toed geckos after Foton-M3 experiments [43]. Neurotrophins can induce cell death via the p75receptor. CD95 mediates receptor-triggered apoptosis. In the gecko study, after the flight on-board Foton-M3, CD95 and p75 immunoreactivity increased in the gecko medial cortex in the flight group compared with both control groups, which provided evidence for the increase in receptor density. The localization of the cell surface transferrin receptor (CD71) was also studied [43]. The transferrin-mediated transport system plays an important role and involves in a wide range of disturbances, including atherosclerosis, arthritis, infection, neurodegeneration, and invasive tumor growth. In the flight group, the immunoreactivity of CD71 was slightly higher than in both control groups. CD71 immunoreactivity distribution (choroids plexus and meninges) might indicate changes in cerebrospinal fluid flow shift after spaceflight.

Taken together, these results provide evidence for signs of the cortex cell alterations and even the damage or predamage status of geckos after Foton-M3. It coincides with data in the literature (including molecular data). It has been known that apoptosis plays an important role in development and tissue homeostasis, but over- or above-levels of normal apoptosis cause a number of functional disorders in humans [70]. Spaceflight condition is reported to induce changes in neuronal cellular organization, including mitochondrial function and oxidative phosphorylation and inflammation which, in turn, could lead nervous cell injury and late neurodegeneration [71]. 

The study of the expression of principal genes encoding several neurotransmitters and genes involved in the neurogenesis and apoptosis revealed close results in the C57Bl/6 male mice brain after the flight on the Bion-M1 satellite [72]. It was shown that genes related to apoptosis were changed in spaceflight. The expression of the antiapoptotic gene *Bcl-xl* has increased in the hippocampus, and decreased in the hypothalamus and striatum, while the expression of the proapoptotic gene *Baxhas* rose in the hippocampus, suggesting the compensation of deleterious spaceflight effects on brain functioning. 

The pattern of the glial fibrillary acidic protein (GFAP)-immunopositive staining in the gecko brain was also studied in the Foton-M2 and Foton-M3 experiments. The GFAP-immunopositive staining in the reptilian brain is represented by radial glia fibers in the forebrain and by radial and astroglia in the cerebellum [73]. A study with GFAP-antibodies revealed that immunoreactivity in the medial and media-dorsal cortex was lower in the brain of flight group geckos after Foton-M2 and Foton-M3 experiments than in the brain of control group geckos in these experiments. The findings coincide with the data on GFAP expression level decreases in the rat hippocampus after microgravity [61]. The cytoskeleton alterations may be one of the causes of apoptosis induced by simulated microgravity [74]. At the same time, no differences were found in the immunoreactivity patterns and intensity of reaction with GFAP antibodies in the cerebellum between the flight and control groups in geckos after the completion of spaceflights on-board Bion-M1 [44]. The increase in the number of astrocytes (astrogliosis) is one of the signs of neurodegenerative disorders [75]. The absence of visible changes in cerebellar glial cells after the spaceflight confirmed the thesis that Purkinje cells had the possibility of a speedy recovery and did not undergo critical changes under spaceflight conditions.

In general, the findings on gecko brains show that the gecko (*Chondrodactylus turneri* GRAY, 1864) is a prospective model in which to study the brain after spaceflights due to the relative simplicity of its organization and, at the same time, its similarities with other vertebrates.

## 4. Reptile Internal Organs in the Spaceflight Experiments

As for the early complex space study of reptile model animals, four turtles (*Testudo horsfieldii*) were used the spaceflight experiment aboard Zond 7 in August 1969. There appeared to be no distinct histochemical differences between the blood elements of the turtles, which had undergone the flight and control condition. Histological and histochemical studies of the myocardium, small intestine, liver, and lung also showed no pathological or metabolic disorder. It is interesting that there were no significant disorders in the alveolar sacs of the upper and middle lung portion and no lesions were observed in the caudal part of the alveolar complex of turtles in the Zond-7 experiment. The epithelium of all lung tissues in all groups appeared normal. The only change revealed in the spaceflight group turtles was the shift in the distribution of cells with small and large nuclei in the respiratory epithelium of the lungs: cells with small nuclei increased to 20% and cells with large nuclei decreased to 2.7%. This tendency was described in all examined tissues; there was a statistically significant increase in the fraction of cells with small nuclei and a decrease in cells with large nuclei in every tissue (cardiac muscle, intestinal epithelium, liver, and lung epithelium) of the animals from the spaceflight group. The authors of the study assumed that this shift in cell distribution after spaceflight conditions reflected changes in the synthesis of nuclear protein [27].

There were no critical or significant differences in the heart, liver, small intestine, pancreas, and spleen of the flight animals comparing to control groups aboard Foton-M2 and Foton-M3 and Bion-M1. The nonpathological and reversible changes revealed in the Foton-M2, Foton-M3 and Bion-M1 experiments are the result of feeding strategy, but not of the spaceflight factors itself. The feeding strategy of experimental animals was one of the main differences in the experimental design aboard Foton-M2, Foton-M3, and Bion-M1. Geckos did not eat or drink aboard Foton-M2, the container in the Foton-M3 experiment was equipped with a water bowl, and geckos were fed with a special space-adapted diet in Bion-M1 (Table 2). Therefore, not all differences between experimental groups in these three experiments were caused by the spaceflight condition itself, such as prolonged weightlessness, but also by the feeding and handled strategy in the three unmanned satellites.

The data taken for Foton-M2 was partly confirmed in the Foton-M3 experiment. The relevant explanation is that during Foton-M3, more homogeneous groups of animals by age, weight, and sex were used. Lower intensity changes in the soft tissues of geckos from Foton-M2 and Foton-M3 experiments could be caused by the shorter duration of the flight and access to water both in the flight and synchronous control groups. The Bion-M1 experiment aboard the biosatellite differed from the Foton-M series by the satellite equipment and internal container environment, as well as by the longer weightlessness time. The changes (still nonpathological) in the internal organs, which might be caused by the spaceflight condition itself, are mainly concerned with the lungs of geckos, while other differences were resolved with metabolism changes associated with the feeding of animals (Table 2).

### 4.1. Liver

After the Bion-M1 experiment, no principal changes were described. Liver weight to animal weight ratio was slightly higher in the flight group in comparison with the control group. The partial loss of glycogen in the hepatocytes of flight group geckos and vasodilation of the capillaries were also described. Three geckos from the flight group had bile outflow difficulty, mainly in the peripheral area, accompanied by necrosis zones. Two geckos had solid adherences in the bile cyst, which also provide evidence for difficulties with bile outflow. After the 16-day flight aboard Foton-M2, the tendency for the trabecular apparatus of the liver to atrophy and hepatocyte degeneration in the outer hepatic zones of the flight group geckos were described. Degraded hepatocytes were seen at the periphery of the liver while the central areas remained intact [29]. It is interesting to note that there were no areas of hepatocytes degradation and zonal destruction at the periphery of the liver in the Foton-M3 12-day flight. A decrease in the quantity of glycogen granules was still found for the Foton-M3 flight and synchronous control group animals compared to basal control group ones, which is a normal reaction for the absence of nutrition.

The closely related general conclusion was made after the analysis of stress-related gene and protein expression in the rat liver after spaceflights [76]. The authors concluded that the effects of spaceflight on the liver might be similar to mild cold stress or fasting. This is interesting, because rodent species in the spaceflight experiment became fatter [77], which was distinct from the gecko model (geckos lost weight in all space experiments aboard both Foton-M and Bion-M1 satellites). In the experiment, rats were analyzed after a 9-day spaceflight, when animals were immobilized and fed with the NASA Experimental Rodent Diet No. 88179 (65% of standard calories).

Also, the alterations in glycogen distribution in the rat liver of the flight and ground control groups were described. Unfortunately, there were no published data on the comparative glycogen quantity (evidenced or not to the glycogen loss) in the rat liver and no information about the rat weights before and after spaceflights. Analysis of the transcriptome showed organ-specific and general reactions for the spaceflight. The study was focused on the comparison of liver and kidney tissue reactions to spaceflight and showed similar gene responses to spaceflight [57]. Nine pathways were upregulated in the liver and downregulated in the kidney. Five of those pathways were related to T cell activation and the others were related to calcium channels, the extracellular matrix structure, heart development, and hormone metabolism. Twelve pathways were upregulated only in the kidney but downregulated in the liver. Changes in cell death pathways, including the overexpression of apoptosis-regulating pathways, were shown in both the kidney and liver. T cell activation was heavily liver-specific in the study. It is likely that the upregulated T cell activation genes in the liver came from the infiltration with inflammatory mononuclear cells. The increased enrichment of genes associated with autophagy in the liver of mice flown for 13.5 days and hepatic negative regulation of protein phosphorylation [78] were observed. In another study [79], the liver, spleen, and thymus from female C57BL/6 mice flown for 13 days were analyzed. Animals from the flight group was reported to have a reduction in liver, spleen, and thymus weights compared with ground controls (tree frog (*Hyla japonica*)); for only two specimens from the flight group, there was also a loss of total protein in the liver after a spaceflight on-board Space Station MIR, while other internal organs remain unchanged [80,81]. It was concluded [79] that exposure to the spaceflight environment can increase anti-inflammatory mechanisms. Increases in liver expression profiles related to fatty acid oxidation with decreases in glycolysis-related profiles were shown for the female C57BL/6J mice flown in space for 13.5 days, which might also correspond to a decrease in immune function during spaceflight [82].

Nevertheless, the spaceflight condition was characterized by a complex changes in gravity, specific air environments, radiation exposure, overloading, and disturbances during takeoff and launch, as well as other stress factors which could cause adaptive changes in metabolism. Therefore, the exact key factor of these molecular alterations in the liver remains unknown, but could correspond to general stress.

### 4.2. Pancreas

Investigations of the pancreas after spaceflights, as well as different experiments staged on Earth, have indicated alterations in the structure and metabolic activity of the pancreas. The most visible changes that appeared in the pancreatic structure after spaceflights is the overfilling of pancreatic vessels: in rats, after a 20.5-day spaceflight on the “Cosmos-690” satellite [83], similar changes were seen in rats after acute and chronic exposure to gravitational overloads [84] and in humans after continuous anti-orthostatic hypokinesia [85]. Moreover, the decreased quantity of exocrine secretory granules in the pancreases of rats after spaceflight with the consequent increase in the biosynthetic activity of exocrine cells during the period of adaptation to gravitation on Earth was highlighted [83]. These data are in line with biochemical studies of the human digestive system during a 1–43 day re-adaptation period after prolonged spaceflights (96, 140, 175, and 185 days), which indicate the increased secretion of the digestive enzymes including pancreatic lipase [86,87].

Biochemical and physiological studies of astronauts during spaceflights suggest the impairment of pancreatic endocrine function. Significant increases in insulin concentration in the plasma and serum has been detected in astronauts in early inflight period [88,89] and at the end of prolonged spaceflights [90,91]. It was also shown that prolonged spaceflights resulted in decreased glucose tolerance [85,90] and increased insulin resistance [91]. The same results demonstrating elevated plasma insulin and C-peptide levels and decreased glucose tolerance were observed in humans after long-term, anti-orthostatic hypokinesia [85,92]. Increased plasma insulin and glucose levels were also reported in animal experiments during spaceflights [93]. Thus, it was suggested that the endocrine pancreas undergoes subclinical diabetogenic changes such as alterations in insulin secretion, insulin sensitivity, and glucose tolerance in microgravity conditions [94].

Molecular data obtained from the genome and proteome analysis of various skeletal muscles of C57BL/N6 mice which were flown on Bion-M1 satellite for 30 days also confirm alterations of carbohydrate metabolism during spaceflight. For example, changes in the expression of genes that are directly or indirectly linked to insulin signaling and sensitivity have been observed in skeletal muscle (longissimus dorsi) of space-flown mice [95]. It was also shown, that soleus proteins involved in glycogen/glucose metabolism were increased in space-flown mice [96].

In the Foton-M2, Foton-M3 and Bion-M1 experiments, histological and immunohistochemical analysis of the pancreas of geckos were performed. In the Foton-M2 and Foton-M3 experiments, the amount of exocrine secretory granules in the pancreas of geckos from the flight and synchronous control groups was greater than in the pancreas of geckos from the basal control groups. Such differences were not observed in the pancreas of geckos from the Bion-M1 satellite. Thus, a reduction in the amount of exocrine secretory granules after spaceflight, which was described for rats [83], was not observed in geckos. It was suggested that the accumulation of pancreatic enzymes in secretory granules was due to the starvation of geckos from the flight and synchronous control groups. In contrast, in the pancreas of geckos from the basal control groups from the Foton-M2 and Foton-M3 experiments as well as in all groups of geckos from the Bion-M1 experiment, which accepted regular feeding, these enzymes were secreted into the intestine during the digestive process and were not accumulated in acinar cells. On the other hand, investigations of the pancreas of geckos during their natural starvation show that exocrine cells undergo vacuolization and apoptosis, but not the accumulation of secretory granules in this period [97]. This difference is possibly connected to the duration of the starvation period: 12 and 16 days in experiments and several months during the hibernation period.

There were also no visible changes in the histological structure of the endocrine pancreas of geckos due to the influence of space factors. Immunohistochemical labeling with antibodies to insulin and glucagon has revealed individual variations in the number of β- and α-cells in the pancreas in animals throughout groups.

### 4.3. Stomach and Small Intestine

There were no principal differences between the flight and control groups in the stomach and intestine structure in the Bion-M1 experiment. After the Foton-M2 flight, dystrophic changes in the small intestine showed an increased number of goblet cells, which occupied the greater part of crypts. Their enhanced secretion was accompanied by cell occurrence in the intestinal lumen. At the same time, the structure and functions of the remaining cylindrical absorptive cells were normal. The major changes include microangiopathy and the disordered morphogenesis of endothelial cells. All of the examined animals had no changed characteristics of the bottom of crypts in Foton-M3.

The only difference revealed in the Foton-M3 experiment is the increased thickness of the small intestine wall in the flight and synchronous control group animals in comparison to the laboratory ones, which is likely caused by the absence of nutrition in the two groups.

### 4.4. Spleen

In the spleen of the Foton-M2 flight group, we found enlarged sinusoids of red pulp surrounded by cells with a granulated cytoplasm (macrophages, granulocytes, and lymphocytes). There were no such accumulations and no enlargements of sinusoids for synchronous control group geckos. In Foton-M3, no such significant differences in the gecko spleens were described. Splenic lymphocyte, monocyte/macrophage, and granulocyte numbers were significantly reduced in the mice after spaceflight [79]. A significant reduction in the expression of key genes in early T cell activation was also shown in the C57BL/6J wild type female mice flown in space for 15 days [98]; the suppression of mouse immune function in spaceflight (as also reported in splenocytes after simulated microgravity) provided evidence for the suppression of mouse immune function in spaceflight. No statistically significant alterations in the spleen cell composition were revealed in the gecko spaceflight experiments. Therefore, to date, the findings in mice could not be confirmed in the gecko model.

### 4.5. Heart

The heart showed a slight tendency of compensatory hypertrophy and high blood inflow, which still remained within the physiological norm in the Foton-M2 experiment flight group animals; there were no signs of compensatory hypertrophy in the Foton-M3 flight group geckos [32].

As it was earlier reported [29], the quantitative analysis of the gecko blood cells revealed a 12% and 40% decrease in erythrocytes and dark nuclear granulocytes, respectively. The number of light nuclear granulocytes remained unchanged.

### 4.6. Lungs

Many lizards, including geckos, possess a single-chambered trabecular parenchymal lung structure. The trabeculae attached peripherally to the lung wall, a system of large polygonal cubicles (ediculae) resulted and such organization was termed edicular to the parenchyma. Secondary and tertiary branching of the trabeculae resulted in a honeycomb-like structure, which was termed faveolar, and the air spaces within it, faveoli. The chamber could also be divided with one or two septas. The complicated lung structure within reptiles is possessed by crocodiles, turtles, and monitor lizards [99,100,101].

The lungs of the flight group geckos in the Bion-M1 experiment were characterized by the high vascular congestion of the capillaries, vasodilation of the large vessels with erythrocyte aggregates, vasodilation of the vascular type of the blood vessels in the acini of pulmonary trabeculae, an increase in the capillary density of the acini of the pulmonary trabeculae, and vacuolization of the capillary endothelial cells [33]. In several cases, an increase in the pulmonary septum quantity of the mural area was described. It is hypothesized that such redistribution of the pulmonary engorgement was caused by microgravity.

The focal endothelial proliferation was revealed by Ki-67 immunohistochemistry, which showed evidence for active angiogenesis. Hypoxia is one of the key factors of the activation of angiogenesis [102]. Hypoxic hypoxia is also characterized by the increase in the pulmonary septum and the vascular congestion [103].

Therefore, it was proposed that the enlargement of the pulmonary vasculature, angiogenesis and the increase in the pulmonary septum surface area in the flight group geckos are the compensatory reaction to the weightlessness as well as to the hypoxia caused by the gas exchange problems in the flight container. The vacuolar changes in the ciliated pulmonary lining cell type and in vascular endothelial cells also were reported for the primitive saccular lungs of two newts after a 15-day spaceflight. These changes were hypothesized as a reaction for chronic mild hypoxia [104].

The main idea resulting from the medical studies during and after space astronaut missions was that the lung was very sensitive to gravity; however, changes in gravity do not result in lasting consequences in its function and it continues to function well [105,106].

For the mouse model, more space studies were performed, including molecular ones. After the 13-day flight on Space Shuttle Endeavour, no abnormality was found in bronchiolar and alveolar epithelium of the C57BL/6Ntac mouse lungs. The loci with the profibrosis-like changes and more abundant collagen accumulation around blood vessels were found in the lungs of several C57BL/6Ntac mice from the flight group. Immunoreactivity of four proteins (MMP-2, CTGF, TGF-1, and NCAM) was revealed to be substantially [107] enhanced by spaceflight; no differences were detected in expression of the MMP-7 and MMP-9 proteins in the same experiment. The analysis of genes associated with the extracellular matrix and adhesion molecules was performed according to quantitative RT-PCR. Fifteen examined genes were shown to be upregulated and 10 were downregulated. As postulated by the authors, these differences could be readily detected shortly after return from spaceflight [107]. The upregulation of four collagen genes from flight samples suggests that collagen synthesis was enhanced, but the downregulation of five of the six MMP genes responsible for the degradation of collagen may attenuate this process. However, there were still no quantitative data on the level of immunoreactivity of the tissues and its correspondence to the gene activity data.

The adhesion molecule gene expression profile was investigated after simulated microgravity in endothelial cell culture [108]. Several shifts in gene expression were detected, but without strong interpretation to the physiological level, these findings remain controversial: “Therefore, we concluded that simulated microgravity per se does not act as an inflammatory factor and does not stimulate the expression of inducible molecules on the EC (endothelial cell) surface.” Another study of the gene expression of cultured retinal cells in simulated microgravity revealed an alteration in cytoskeletal-related proteins and some cellular adhesion gene expression, but this was also without histological or physiological level implications [109]. The resulting effects and functional significance of the adaptation of the space conditions of these molecular changes remain unclear. Also, cytoskeletal changes as a rapid answer to changing gravity during parabolic flights and hyper-gravity was shown for cell culture [109]. Nevertheless, the experimental data received for the cell culture in short-term microgravity might be nonapplicable for the in vivo tissue at the organism level. Further physiological, histological and immunohistochemical data are needed to understand the real implication of the molecular findings in spaceflight alterations and adaptations.

## 5. Weightlessness Effects on the Skeletal Bone of Reptiles in the Prolonged Space Experiments

### 5.1. X-Ray Fluorescence Analysis (XFA), Scanning Electron Microscopy, and Tomographic Studies

Bones are complex tissue with the continuous remodeling which it undergoes in physiological conditions [110]. Normally in human adults on Earth 20%–30% of the bone is replaced each year [111]. Studies of the influence of spaceflight factors on the human and model animal skeletal bones have been conducted for a long time, and remain vital due to the serious problems attended astronauts in spaceflights [111,112,113]. Average bone loss during spaceflight is ~1%–2% per month with the most effect in the weight-bearing bones of the legs and spine. There are reported cases of individuals having lost as much as 20% of bone mass throughout their lower extremities after six months in orbit. To date there is no indication that this bone loss abates with longer flights, but it is known that bone loss continues for several months after returning to Earth [111]. Such considerable bone loss may lead not only to increase in fracture risk, but also to renal stone formation and changes in bone marrow [111]. Another consequence of long-duration spaceflights is deficits in the function of cartilage, tendons, and vertebral disks [114], with subsequently increased disc herniation risk in astronauts [113].

Astronauts should regularly perform weight loading physical exercises with the purpose to simulate the Earth gravity. However, only physical activity could not prevent the muscle and bone loss during spaceflight [111]. Whether it depends solely on the reduced mechanical loading in weightlessness or are compounded by fluid shifts, altered tissue blood flow, radiation exposure, and altered nutritional status is not yet well defined [115]. Completely effective countermeasures for mitigating the effects of weightlessness on humans remain unknown [116], although the problem is also evident on Earth. Clément [111] reported that the average decrease in bone mass is ~1–2% per decade after the age of 30 years. For women this rate increases to 1–2% per year somewhere between three and eight years after menopause. Osteoporosis is a bone disease in which the bone mass is reduced by 0.5 to 2.0% per year in G_1_ (approximately the same the loss is seen in G_0_ per month). It often leads to fractures without any precursor symptoms. However the bone loss observed after a spaceflight of a few months corresponds to that of several years on the ground [111]. Therefore, the problem of bone loss is important both on Earth and in spaceflights.

Bone loss in spaceflight was investigated in humans [111,117,118] and animal models, including the most popular experimental objects—mice [113,119,120,121,122]. The main method in these studies was computed microtomography (μCT). This approach has two problems: the small statistical sample size of almost all human spaceflight studies and the small size of the observed bone structures compared with the spatial resolution of the available imaging devices [110,118]. One of the ways to overcome these obstacles is research on animals that have been carried out both on lower vertebrates [123,124,125], as well as mammals and primary rodents [126]. However, the use of these model objects is complicated by the fact that bones grow throughout life in rodents, while this process is restricted by puberty in humans [111].

Another problem is flotation experienced by vertebrates in spaceflights, which produce stress in animals and restricts the ability to move normally. Gecko bones also grow throughout life, but it is the unique natural model objects, that retain practically normal locomotion and behavior in weightlessness [6,32,41,42]. Reptiles possess a skeletal bone organization that is typical for vertebrates, consisting of the axial, limb skeleton, and neuro- and viscerocranium. The mineral metabolism of reptile bones is closer to that of mammalian one.

Reptiles (turtles) were the orbital experiments subjects in the USSR. Mineralization, the mineral to organic ratio and bone density, as well as blood vessels quantities in the histological sections of the epiphysis and diaphysis of the humerus and femur were investigated. There were no changes in the bone structure between control and flight groups after 19 and 22 days of flight, and there was only a tendency towards demineralization in the case of 60- and 90-day flights. Also, there was no dependence of mineralization on the weight of animals was reported [28].

For the complex skeletal bone investigation, the axial, limb, and visceral bone elements of thick-toed geckos were chosen in the Foton-M and Bion-M experiments [6,32,33,41,42]. The results of the gecko model research, comparing them with the literature data obtained for other model objects are provided.

### 5.2. Limb and Visceral Bones of Geckos After 12–30-Day Spaceflights

In the Russian–American experiment onboard Foton-M2 (31.05.2005–16.06.2005) skeletal bones of geckos were independently studied in Russian and USA laboratories [29,30].

The geckos in the preflight control experiments and 16-day Foton-M2 spaceflight without food and water showed no obvious negative health effects. However, detailed μCT analysis of bone mass and architecture revealed significant loss of cancellous bone in the distal femur and humerus both in the synchronous control and spaceflight animals relative to basal control group. In addition, the shift in the DNA content from G2 and S to G1 cell cycles was observed in the 30h postexperimental liver tissue both in spaceflight and delayed synchronous control groups (DSC) [30]. Nondestructive three-dimensional evaluation of bone volume and architecture was used. Gecko humeri and femora were scanned at a voxel size of 12 μm using a Scanco μCT40 scanner (Scanco Medical AG, Brüttisellen, Switzerland) and evaluated at a threshold of 265 [30]. Total bone measurements were performed and cancellous bone was evaluated. The ratio of bone volume to total volume in spaceflight and synchronous controls decreased dramatically relative to basal controls [30]. Specifically, the cancellous region of the distal femur lost nearly 50% bone mineral in spaceflight as well as in synchronous controls. The humerus results, however, were only significant with regard to bone volume, which decreased by approximately 45% in both the DSC and flight groups. The authors explained the data obtained by the assumption that housing conditions were not conducive to good bone health [30]. Thus, the matrix decrease was observed in both the flight and DSC groups. It can be assumed that the decrease was caused by the absence of food and water and the volume of the compact container, but not by the space condition itself.

**Figure 2:**
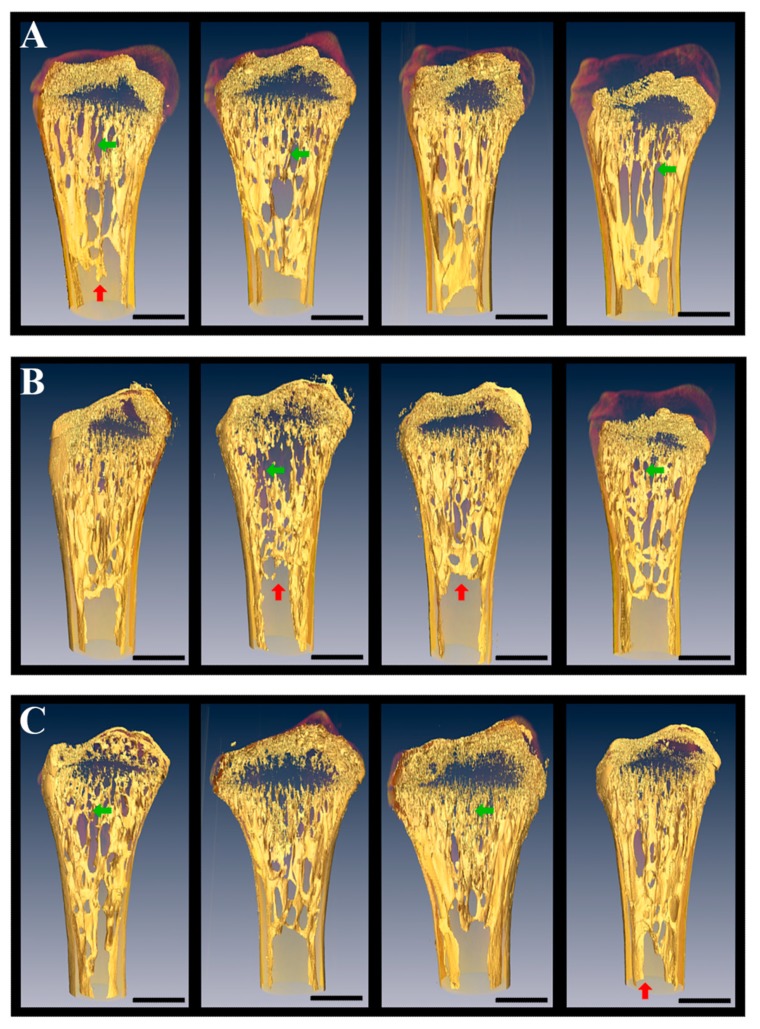
Distal parts of thick-toed gecko femur were examined by SkyScan-1172 microtomograph after the orbital experiment onboard Foton-M3 USC (unmanned spacecraft). Tomographic slices with cancellous bone. (**A**) 12-day flight, (**B**) delayed synchronous control, and (**C**) basal control. The red arrows indicate the different axial extent of the cancellous bone, the green arrows indicate the different concentration of trabeculae. Bar: (**A,B,C**) 1 mm.

In another study, it was examined whether the biomechanical interaction of the gecko limbs with the container wall influence gecko adaptation to the spaceflight condition. Anatomical and histological investigation of the humerus and limb fingers reveals no alteration on the microscopic level. X-ray μCT at different wavelengths, scanning electron microscopy, and X-ray fluorescence analysis were used for the bones study in the Foton-M2 and Foton-M3 experiments. Femur, tibia and fibula were examined by SkyScan-1172 microtomograph with resolution of 8 to 32 μm and by means of a device built by the FSRC “Crystallography and Photonics” with resolution of 9 to 13 μm [29,46]. These approaches allowed qualitative and quantitative comparison of skeletons of flight and control animals. μCT did not reveal any demineralization of skeleton in microgravity. Quantitative assessment of the changes was not carried out, although the individual features of the structure of the femur—which may not be associated with the influence of weightlessness—are seen (Figure 2).

Also the analysis revealed no alteration in the entire volume of the extracellular matrix in the flight and synchronous control groups. Nevertheless, both were lower than in the basal control group. These results completely correspond to the above-described data [30], in agreement with the published data showing that cancellous bone was more sensitive to the effects of weightlessness than the bone cortex [113]. At the same time, there was no cancellous bone loss revealed in geckos after the 12-day spaceflight, while rats were reported to show a loss [113].

The 3D-reconstruction and quantitative analysis of the extracellular matrix were performed on gecko tibias. μCT with the Cu- and Mo-tubes produced secondary X-ray of the different wavelengths [31,46], which was more appropriate than the SkyScan-1172 and Scanco Medical AG using wideband X-ray source. Fibroblast collagen, ligaments, and aponeuroses surrounding the bones structures could distort the X-ray μCT results. To avoid the artifacts of the volume measuring originating from the aforementioned tissue monochromator, enable to select narrow bands of X-ray range was used. This method confirmed the absence of alterations in the limb bones between the flight and synchronous control groups [46].

Thus, two independent research teams with the four types of μCT provide the same results, which show evidence for the possible positive influence of the attachment ability of gecko on the mineral metabolism and decreased demineralization in weightlessness.

It is known that the bone loss induced by weightlessness environment during spaceflight is site-specific [122]. Usually alterations concerned in the weight-bearing bones which provide a rigid support for the body in Earth’s gravity [116]. That is why femur and tibia were studied for the Foton-M2 and M3. Similar studies were performed for Bion-M1 mice by several independent research teams [122]. The mature male C57/BL6 mice femur after the 30-day Bion-M1 mission and 8-day recovery period were studied. The decreasing of the trabecular bone volume (−64% vs. Habitat Control), the bone resorption dramatically increasing (+140% vs. Habitat Control), and marrow adiposity invasion inducing were shown in the femur metaphysis after spaceflight. The cortical thinning associated with periosteal resorption was observed in the diaphysis. Reduced volume and a more spherical shape were demonstrated in osteocyte lacunae and empty lacunae were highly increased in the flight group (+344% vs. habitat control). Mechanical cortical properties, including hardness and modulus, were shown to locally decrease after spaceflight, whereas the mineral characteristics and collagen maturity remained unaffected. Decreasing of the overall bone volume of the vertebrae was observed after spaceflight, also as alterations of the modulus in the periphery of the trabecular struts. Normalized osteoclastic activity and an increasing of osteoblast count was found, nevertheless no bone recovery was reported 8 days after landing. In conclusion, it was speculated that osteocyte death had been induced by the spaceflight, which could lead in the bone resorption, bone mass change, and microstructural deterioration. There was also hypothesized that osteocyte cell death, lacunae mineralization, and fatty marrow, which are hallmarks of aging, may impede tissue maintenance and repair [122]. The absence of the Ca loss in the 12–16-day flown geckos may concern with the insufficient flight periods, the presence of a support reaction or by the features of geckos metabolism.

**Figure 3:**
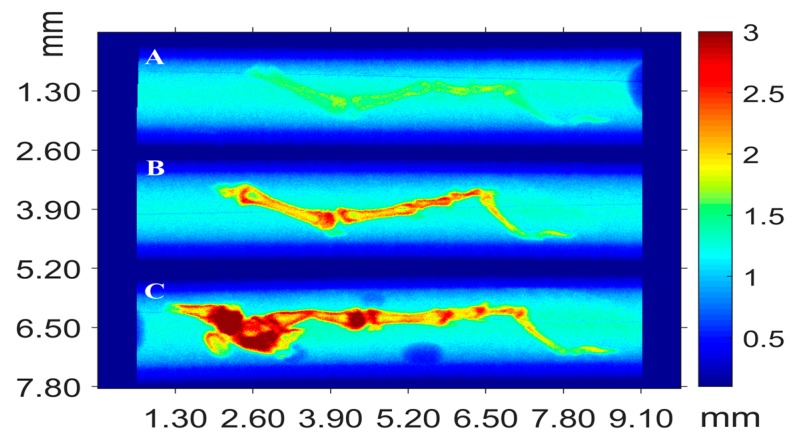
X-ray μCT projections of the thick-toed gecko phalangeal bones. (**A**) Flight group gecko toe, average absorption for the sample—1.429. (**B**) Delayed synchronous control group gecko toe, average absorption for the sample—1.700. (**C**) Basal control group gecko toe, average absorption for the sample—3.136. Extracellular matrix density is colored in the right scale: the densest areas are red-orange. In the toe of gecko exposed to weightlessness twofold low density in comparison with the laboratory control is seen.

Toes of geckos were investigated to evaluate the adaptive processes distribution with the 3D bone organization after the 16-days spaceflight onboard Foton-M2. X-ray μCT showed that in some cases absorption coefficient of the phalangeal bones of the geckos from the basal control group exceeded those of the flight and synchronous control groups (Figure 3). However, no significant intergroup difference was found. Perhaps, phalangeal bones demonstrated more rapid reaction to the weightlessness than the main limb bones, but more research is needed to verify this.

The visceral skeleton is only indirectly involved in the body supporting and moving the body; nevertheless, in this case, a demineralization problem is also possible. Teethed lower jaw (mandible bone) of geckos were used for the investigation of the visceral skeleton demineralization in weightlessness after the 16 days spaceflight (Foton-M2) [29,45]. The lower jaws were examined by the same methods as the limbs, with similar results. [45].

Demineralization of the limb and mandible bones was revealed by the X-ray μCT only in the flight and synchronous control groups, providing evidence that it was caused by the experimental conditions, including food and water absence, but not by the weightlessness itself.

### 5.3. Caudal Vertebrae of Geckos after 12–30-Day Spaceflight

The caudal vertebra of geckos had almost no loading in weightlessness—the behavioral study of the video recording material in microgravity showed only rare tail use with the purposes of obtaining additional mechanical information, of communication and during short-term forced flotations [26,33,42]. The caudal vertebrae were investigated after the prolonged spaceflight with the electron microscopy, X-ray fluorescence analysis, energy-dispersive X-ray analyzer (EDAX), X-ray μCT and Mediana, and RT-MT synchrotron stations [45].

#### 5.3.1. X-ray Fluorescence Analysis (XFA)

XFA is a modern spectroscopic nondestructive method which allows one to detect almost all elements of the periodic table with mass fractions from 10 to 4 to 100% (depending on the experimental conditions). This method is based on the detection and subsequent analysis of the characteristic fluorescence spectrum produced by the X-ray irradiation of the sample. The experimental spectra were processed by the PyMCA program (European Synchrotron Radiation Facility); using this program, the composition of the samples was qualitatively analyzed and preliminary quantitative estimates were made. Four samples were analyzed: S3 and F3 from the Foton-M2 experiment and S1 and F1 from the Foton-M3 experiment (F and S are the flight group and the DSC, respectively) [45].

The investigations performed showed that the differences in the elemental compositions of the flight and synchronous samples are small within each series and can be related to the individual features of the samples. The following elements were found in the composition of the Foton-M2 samples; Ca, Zn, Fe, Sr, Cu, As, Pb, Br, and S. Flight and synchronous samples differ in the fluorescence yield intensities from Cu, Pb, S, and Sr. The following elements were found in the composition of Foton-M3 samples Ca, Zn, Fe, Sr, Cu, and S. The compositions of flight and synchronous samples differ in the fluorescence yield intensity from Fe, Zn, and Sr. A comparison of the results for the Foton-M2 and Foton-M3 series showed the following features.

(i) An increase in the signal from Ca and Sr for the samples of series Foton-M3.
(ii) The absence of elements As, Pb, and Br in the composition of the samples of series Foton-M3.
(iii) A decrease in the signal intensity from Cu in the samples of series Foton-M3.

This difference in sample compositions may be caused by the specific features of the orbital experiments and the methods of sample preparation and storage.

#### 5.3.2. Scanning Electron Microscopy

The elemental compositions of samples S3 (Foton-M2 series) and S1 (Foton-M3 series) were additionally analyzed by scanning electron microscopy. The results obtained revealed the presence of C, O, Na, Al, Ca, K, Mg, P, and Cl. A difference in the compositions of the samples was found, which may be due to the surface microinhomogeneities caused by individual features. The fluorescence yield intensity from heavier elements (with energies above 4 keV) was at the sensitivity limit of the detection system. Such a significant difference in the XFA and SEM data on the chemical composition of the samples can be explained by the nonuniform distribution of elements in the samples; i.e., one may conclude that heavier elements are concentrated in the sample bulk, which is confirmed by X-ray microtomography data (see below).

#### 5.3.3. X-ray μCT

Tomographic studies of the bulk structure of the bone tissue of animals after their stay under conditions of microgravity onboard Foton-M2 and M3 (16- and 12-days, respectively) were carried out with a laboratory X-ray microtomograph and Mediana and RT-MT synchrotron stations. Peripheral portions of the samples contained no elements with atomic numbers above 20 (which corresponds to calcium) or a concentration below the sensitivity limit. This is in good agreement with the SEM data. In addition, the X-ray absorption coefficient was found to significantly decrease (by a factor of 3–5) in the samples of the Foton-M2 series in comparison with that for the samples of the Foton-M3 series.

**Figure 4:**
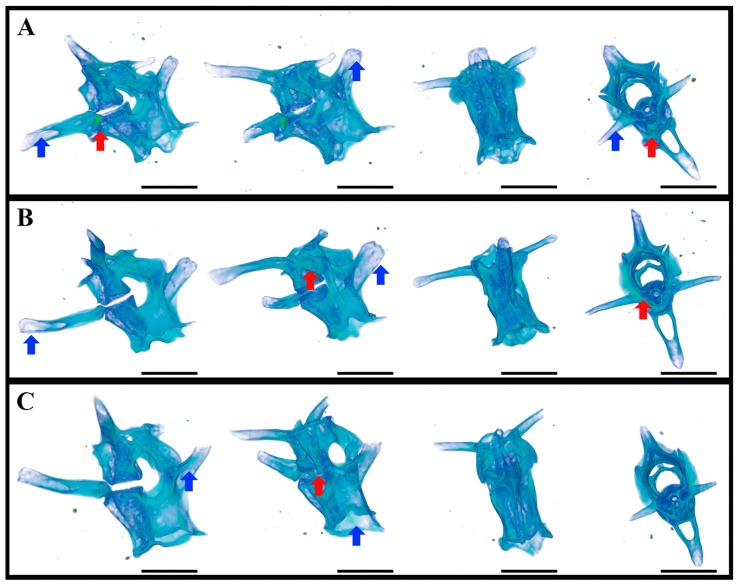
X-ray μCT of the thick-toed gecko proximal vertebrae bone results (Bion-M1). (**A**) terrarium control group gecko. (**B**) Delayed synchronous control group gecko. (**C**) Flight group gecko. Extracellular matrix alterations are well seen in the high-density areas (green colored, red arrows). Extracellular matrix density in the low dense areas (blue colored, blue arrows) remains unchanged. These results show that the high dense bone areas are the starting points of the demineralization process. Bar: (**A,B,C**) 1 mm.

X-ray μCT also revealed no significant alterations between the three experimental groups in the Bion-M1 experiment (Figure 4).

A complex study of the influence of microgravity on the processes occurring in the elements of locomotor apparatus (proximal tail vertebrae) of thick-toed gecko was performed for the first time. The results of conventional histological studies were compared with the X-ray μCT, XFA, and SEM data. The absorption density of these vertebrae was found to vary significantly, depending on the experimental conditions and methods of sample preparation. Regions characterized by elevated density were found near the central vertebral canal. It should be noted that the ratios of absorbance in different bone tissue regions changes with a change in the probe X-ray wavelength. This circumstance indirectly indicates that the elemental composition is distributed nonuniformly over the bone volume. In this context, the XFA data were found to correlate with the results obtained by μCT. Based on the XFA data, the presence of a number of heavy elements (Fe, Ni, Cu, Zn, Br, and Sr) in the structures under consideration was established for the first time; their distribution was found to be nonuniform. A comparison of the SEM and XFA data shows that these elements are located in the bone tissue bulk (apparently at a distance larger than 10 μm from the surface), which is also in agreement with the X-ray microtomography data. It is reasonable to suggest that the revealed regions of elevated (electron) density can be primary ossification centers. The study must be continued to establish if the presence of strongly absorbing elements and their nonuniform distribution characterizes only the above-mentioned vertebrae and if these features are typical of the other parts of locomotor apparatus of thick-toed gecko [45,46].

The above-mentioned results can be compared with the data obtained by the other research teams taking part in the project, including the study of the proximal caudal vertebrae of mice after the same experiment as described above (30-day flight to the Bion-M1 satellite) [113]. The motion segments of mouse spine using SCANCO Evaluation Program v6.5-3 in the Bion-M1 experiment were studied. Bone volume fraction (BV/TV), bone mineral density (BMD), and trabecular microarchitecture (trabecular number, thickness, and spacing) on each whole motion segment of mouse spine were quantified. The μCT results support bone weakening as the cause for failure mechanism differences observed between flight and control mice. For the trabecular VOI (volume of interest) adjacent to the growth plate, the authors found that spaceflight significantly reduced BV/TV, BMD, and trabecular thickness. They revealed the 18% reduction in BMD. The trabecular thickness was the most significantly reduced parameter in the study. It was shown to decrease by 14% for the trabecular VOI and 9% for the entire vertebra. The data are consistent with the report of decreasing trabecular thickness in hindlimb unloading model of rat tibia [127]. The absence of the significant changes in other nonspecific to the trabeculae bone parameters of the entire vertebra was explained by the higher bone turnover in the trabecular than cortical bone [113,128].

No alterations were revealed in the proximal vertebras of geckos in the same experiment (30 days flight onboard Bion-M1) using X-ray μCT, which may concern with the peculiarities of the gecko biology and physiology [33].

### 5.4. Cellular and Molecular Bone Studies after the Spaceflights

It is interesting to compare the data on the effect of weightlessness on the bones of the skeleton of humans and animals, obtained by physical methods, with the currently available research results at the cellular and molecular levels. It was shown, that the osteoblasts in cell culture have the following alterations after five days of microgravity: cytoskeleton changes, including shorter and wavier microtubules and thinner cortical actin and stress fibers, also smaller and fewer focal adhesions. Extended cell shapes and significantly more disrupted and fragmented or condensed nuclei were observed in the osteoblasts after spaceflight [129].

Later, altered gene expression levels of 81 mRNA following spaceflight was reported on the material of the eight mice after the 15-day STS-131 space shuttle mission [120]. Using μCT it was shown that pelvis decreased in bone volume fraction (BV/TV) of 6.29%, and bone thickness of 11.91%. Osteoclastic bone degeneration in microgravity was indicated by increasing TRAP-positive osteoclast-covered trabecular bone surfaces by 170% (*p* = 0.004). Lacunar osteolysis, including increases in cross-sectional area (+17%, *p* = 0.022), perimeter (+14%, *p* = 0.008), and canalicular diameter (+6%, *p* = 0.037) was revealed by high-resolution X-ray nanoCT studies. Osteocytic osteolysis was also evidenced by the RT-qPCR data on the expression of matrix metalloproteinases (MMP) 1, 3, and 10 in bone, which were shown to upregulate in microgravity (*p* = 0.01). Furthermore, increasing (*p* = 0.01) of the *CDKN1a/p21* expression localized to osteoblasts was reported, which could affect inhibitory on the cell cycle during tissue regeneration and confer apoptosis resistance. Thus, it was shown, that the microgravity induced *CDKN1a/p21*-mediated osteogenic cell cycle arrest and osteocytic osteolysis. In addition, the *Trp53* apoptosis inducer was downregulated by 21.54-fold (*p* = 0.01). In conclusion, the pelvic and femoral region of the mouse skeleton was identified as an active site of rapid bone loss in microgravity, which was not limited to the osteoclastic degradation [120].

One of the greatest achievements of the molecular direction in the past decade—the clarification of the role of the sclerostin and Wnt-β-catenin signaling for disuse bone loss [115]. Sclerostin is the osteocyte protein product of the gene *Sost*. At the beginning of the current century it was revealed that deletions in the *Sost* gene lead to bone overgrowth of the skull, mandible, and/or hand digits [130,131]. The next step was identification of the protein product of *Sost*, sclerostin, which inhibits binding of Wnt to the Lrp 5/6 receptor [132], which downregulates activity of the Wnt-β-catenin signaling pathway, and therefore osteoblast function. It was hypothesized [133,134] that some bone cells constitutively produce an inhibiting signal for bone formation by osteoblasts. Mechanical loading causes an increase in osteoblast activity, which, in turn, reduces the expression of the signal, hence allowing site-specific upregulation of bone formation. Mesenchymal stem cells into the osteoblastic cell line differentiation is modulated by the Wnt-β-catenin signaling, which may indirectly impact osteoclast function by regulating osteoblast [135] and osteocyte [136] release of osteoprotogerin—the receptor for RANK that inhibits osteoclast differentiation. Also, increasing sclerostin in serum after 60 days of a 90-day bed rest [137], as well as in bedridden stroke patients [138], suggests this mechanism to be relevant for extended microgravity effect. These results were also confirmed by the several studies on animals [139,140,141]. Little to no loss of cancellous or cortical bone was demonstrated by the *Sost*−/− mice in response in the hindlimb unloading [142] or muscle paralysis induced by botulinum toxin experiments [143].

Dickkopf-1 (Dkk1), Wise, and Fzd-related proteins are also regulators of Wnt signaling acting at the Lrp 5/6 receptor [144], which may increase in the absence of sclerostin. This upregulation may provide an explanation for the enhancing of bone formation in response to cyclic axial loading of limb bone in anesthetized *Sost*−/− mice as compared to the wild type control [143].

Changes in expression of multiple genes in response to spaceflight or hindlimb unloading were reported [120]. A list of genetic models tested for their response to disuse bone loss, including several connected with mechanotransduction (β-1 integrin) or cell adhesion to mineralized matrix (cas-interacting zinc finger protein, osteopontin) was reviewed [115].

Further investigations are needed to explain how these various molecules and their signaling pathways might contribute to the alterations in skeletal integrity during disuse or the prolonged nonweight-bearing in the space environment [115].

Despite the progress made in clarifying the molecular mechanisms, the initial action of microgravity has not been identified [124]. Medaka transgenic lines expressing osteoblast and osteoclast-specific promoter-driven GFP and DsRed were analyzed by live imaging of animals during a space mission followed by transcriptome analysis. Significantly enhancing of the intensity of osterix- or osteocalcin-DsRed fluorescence in pharyngeal bones one day after launch was revealed, which continued for eight or five days. Highly increasing of the signals of *TRAP*-GFP and *MMP9*-DsRed in osteoclasts was reported at days 4 and 6 after launch. Pharyngeal bones of juvenile fish HiSeq showed up-regulation of 2 osteoblast- and 3 osteoclast- related genes at the day 2 after launch. Fish whole-body gene ontology analysis showed significantly enhancing of the transcription of genes of the “nucleus” category. Transcription regulators were more upregulated on day 2 than day 6. Five genes (*c-fos, jun-B-like, pai-1, ddit4*, and *tsc22d3*) were identified as upregulated in the whole-body at days 2 and 6, and in the pharyngeal bone at day 2. This results evidence that dynamic alteration of gene expression levels in osteoblasts and osteoclasts is induced by microgravity [124].

Such destructive changes observed for the rat and mouse bones were not revealed for the geckos. The axial skeleton study of the thick-toed geckos showed the same results as limb and visceral skeletal ones. Inconspicuous demineralization was observed for the mostly Ca- and F-rich bone areas. These bone reorganizations didn’t consume the main skeletal bone parts. The influence of weightlessness during 12–30-day spaceflights didn’t significantly affect the mineral metabolism of the thick-toed geckos. Foton-M series and Bion-M1 bone experiments evidence that the ability to attach to the surfaces and retain normal locomotion (perhaps with other physiological and metabolic gecko features) provide stable mineral metabolism and avoids demineralization in the gecko skeletal bones in prolonged weightlessness. The mechanisms underlying this result remain unclear and further orbital experiments and molecular and genetic investigations are requested.

## 6. Conclusions

Reptiles are rare objects for the spaceflight researches. Since the Foton-M2, geckos were used for the prolonged space research aboard unmanned satellites. The advantage of the gecko model is the gecko ability to remain attached the surfaces during the weightlessness. Geckos avoid floatation in the spaceflight condition thus keeping normal behavior pattern, minimalizing stress. Also the thick-toed geckos demonstrate the ability of their nervous system to coordinate the information from the vestibular apparatus with tactile and visual signals in the weightlessness and possess generally high adaptation to the spaceflight conditions.

Demineralization and neuromuscular problems in the absence of the support reaction are also reported for mammals including human. Foton-M series and Bion-M1 bone experiments allow us to speculate that the ability to attach to the surfaces and to retain normal locomotion, perhaps with other physiological and metabolic gecko features, provide stable mineral metabolism and avoiding demineralization in the gecko skeletal bones in prolonged weightlessness. Thus geckos are useful animal model for orbital experiments. The geckos also possess wide range of the species of different weights and biology, including low-depending from water animals, which allows choosing optimal specimens for the orbital experiments aboard unmanned satellites and space station. 

No critical or significant differences in heart, liver, small intestine, pancreas, and spleen of the flight thick-toed geckos aboard Foton-M2 and M3 and Bion-M1 were observed. The major nonpathological and reversible changes revealed in these experiments are the result of feeding strategy, but not of the spaceflight factors itself. Nevertheless, the spaceflight condition was characterized by a complex changes in gravity, specific air environment, radiation exposure, overloading, and disturbances during take-off and launching, as well as other stress factors, which could cause adaptive changes in metabolism. The molecular mechanism of these adaptations caused by the weightlessness and other flight specific condition remain unclear. The published molecular data are restricted and more physiological, histological and immunohistochemical results are needed to understand the real implication of the molecular findings in the spaceflight alterations and adaptations.

## Figures and Tables

**Table 1:** The main orbital and parabolic flight experiments with reptiles.

Parabolic Flight		Orbital Flight					
		Zond 5, Zond 7	Series of Orbital Flights (USSR)	Foton-M2	Foton-M3	Bion-M1	Foton-M4
7 s	20–25 s	7 days	19–90 days	16 days	12 days	30 days	45.5 days
*Trachemys ornata, Hydromedusa tectifera* [25]	Reptiles (21 species), including geckos [26]	*Testudo horsfieldii* [27]	*Testudo horsfieldii* [28]	*Chondrodactylus turneri* [29,30]	*Chondrodactylus turneri* [31,32]	*Chondrodactylus turneri* [33]	*Phelsuma ornata* [34]
Behavior	Behavior	Blood, internal organs, peripheral nervous system	Skeletal bones	Behavior, blood, internal organs, central nervous system, setae, skeletal bones, excrements	Behavior, internal organs, central nervous system, setae, skeletal bones, excrements	Behavior, internal organs, central nervous system, setae, skeletal bones	Behavior

**Table 2:** The main parameters of the gecko orbital experiments.

Parameter	Foton-M2, 2005, 16-Day Flight	Foton-M3, 2007, 16-Day Flight	Bion-M1, 2013, 30-Day Flight	Foton-M4, 2014, 44.5-Day Flight
Species	Thick-toed geckos *(Chondrodactylus turneri* GRAY, 1864)	Thick-toed geckos *(Chondrodactylus turneri* GRAY, 1864)	Thick-toed geckos *(Chondrodactylus turneri* GRAY, 1864)	Thick-toed geckos *(Phelsuma ornata* GRAY, 1825)
Geckos in flight group	5 (1 group from 5 geckos)	5 (1 group from 5 geckos)	15 (3 groups, each from 5 geckos)	5 (1 group from 5 geckos)
Weight ^1^ (g)	F—16.9 *, M—24,7 *	17.0 *	20,2 *	F—3.8 *, M—4,3 *
Weight ^1^ (g) before flight/after flight	F—16.9/15.35 *, M—24.7/21.8 *	17.0/15.6 *	19.8/18.8 *.	F—3.8/2.3 *, M—4.3/3.0 *
Longevity in terrarium	4–6 * years			
Type of activity	Night			Day
Landing-fixation period (hours)	32.0	13.5	13.5	6.1
Temperature (C^0^)	16.5	20.9	19.0	21.1
Air regeneration	no	no	yes	no
Water supply	no	yes	yes	yes
Nutrition	no	no	yes	yes

*—average; ^1^— F—average data for 4 females; M—data for 1 male where applicable.
